# The Acute Effect of Hot Water Immersion on Cardiac Function in Individuals with Cervical Spinal Cord Injury

**DOI:** 10.3390/jcm13247593

**Published:** 2024-12-13

**Authors:** Ken Kouda, Motohiko Banno, Yasunori Umemoto, Tokio Kinoshita, Yukihide Nishimura, Yukio Mikami, Toshikazu Kubo, Fumihiro Tajima

**Affiliations:** 1 Department of Rehabilitation Medicine, Wakayama Medical University School of Medicine, 811-1 Kimiidera, Wakayama 641-8510, Japan; banno@wakayama-med.ac.jp (M.B.); tokio@wakayama-med.ac.jp (T.K.); tkubo@koto.kpu-m.ac.jp (T.K.); tajibun@gmail.com (F.T.); 2 Research Center of Sports Medicine and Balneology, Nachikatsuura Balneologic Town Hospital, Wakayama 649-5331, Japan; 3 Department of Rehabilitation Medicine, Yokohama City University School of Medicine, Kanagawa 236-0004, Japan; umemoto.yas.ko@yokohama-cu.ac.jp; 4 Department of Rehabilitation Medicine, Iwate Medical University School of Medicine, Iwate 028-3694, Japan; ynishi@iwate-med.ac.jp; 5 Department of Rehabilitation Medicine, Hiroshima University Hospital, Hiroshima 734-8551, Japan; mikamiy@hiroshima-u.ac.jp; 6 School of Medicine, Kyoto Prefectural University of Medicine, Kyoto 602-8566, Japan; 7 Chuzan Hospital, Okinawa 904-2151, Japan

**Keywords:** head-out immersion, heat stress, spinal cord injury, sympathetic nerve dysfunction, cardiac function

## Abstract

**Background/Objectives:** Thermotherapy is expected to assist in the prevention of arteriosclerosis and cardiovascular disease in individuals with spinal cord injuries. This study aimed to investigate the impact and underlying mechanisms of whole-body heat stress on cardiac function in patients with cervical spinal cord injury (CSCI) and healthy controls using head-out hot water immersion (HHWI). **Methods:** Eight male patients with complete motor CSCI and nine healthy controls were recruited. Participants were immersed for 60 min in water set at 2 °C above the resting esophageal temperature. Esophageal temperature, heart rate, and arterial pressure were monitored throughout the experiment. Before and after HHWI, echocardiography was used to measure indices of left ventricular diastolic capacity (E, E′, and A), left atrial contractility (A and A′), and left ventricular contractility [S′ and isovolumic acceleration (IVA)]. **Results:** Both groups exhibited an increase in body temperature and heart rate, while blood pressure remained stable. In the control group, there was a significant increase in E (67.0 ± 22.6 to 89.1 ± 13.6), E′ (9.5 ± 3.8 to 15.1 ± 4.1), A (50.0 ± 15.2 to 75.8 ± 18.2), A′ (8.1 ± 1.6 to 14.8 ± 5.9), S′ (8.7 ± 1.4 to 15.1 ± 4.5) and isovolumic acceleration (IVA) (104.2 ± 14.7 to 151.1 ± 20.6). In the CSCI group, only A (49.5 ± 9.9 to 56.9 ± 10.9) and IVA (94.4 ± 27.2 to 134.7 ± 27.7) showed a significant change. **Conclusions:** In the control group, heat stress increased left atrial contractility, left ventricular dilatation, and left ventricular contractility, while in patients with CSCI, left atrial contractility and left ventricular contractility improved, but there was no improvement in left ventricular diastolic function. This discrepancy in the impact of HHWI on cardiac function suggests that the sympathetic nervous system predominantly influences left ventricular dilatation during whole-body heat stress. However, other factors may also contribute to left atrial and ventricular contractility.

## 1. Introduction

Individuals with spinal cord injury (SCI) exhibit elevated levels of C-reactive protein and tumor necrosis factor-alpha in the blood, leading to a state of chronic low-grade inflammation [1]. This chronic inflammatory state contributes to the progression of atherosclerosis, which increases the incidence of cardiac disease and is a cause of death in individuals with cervical spinal cord injury (CSCI). The incidence of cardiac disease is higher in patients with CSCI than in healthy individuals. Given the current increase in life expectancy among individuals with SCI, addressing the issue of cardiac disease in this population is imperative.

Heat stress places a considerable burden on the human cardiovascular system, and bathing is not advised, particularly in patients with severe heart failure [2]. Recently, however, there has been growing interest in the potential of warm baths as a non-pharmacological treatment for chronic heart failure [3,4]. Repeated sauna treatment, referred to as Waon therapy, has been observed to ameliorate congestion and improve vascular endothelial function, cardiac function, and exercise tolerance in patients with chronic heart failure [4,5]. Moreover, a number of thermal therapies have been demonstrated to be efficacious in prolonging healthy life expectancy. This includes the potential benefit of sauna bathing in reducing cardiovascular mortality [6,7]. Thus, this approach may prove efficacious in mitigating the progression of arteriosclerosis and heart disease in individuals with SCI.

Whole-body heat stress affecting the cardiovascular system has been demonstrated to decrease central and left ventricular systolic blood volumes and ventricular filling pressure through peripheral vasodilation in healthy individuals [8]. In the absence of compensation, a reduction in the ventricular filling pressure results in a decrease in stroke volume. However, under whole-body heat stress, this does not occur, and the cardiac output increases due to an increase in systolic cardiac function and the maintenance of diastolic function [9]. Furthermore, systemic vascular resistance is diminished, thereby maintaining the arterial pressure at a virtually unaltered level. This improved cardiac output is primarily attributable to an increase in heart rate with no discernible change or only a slight increase or decrease in stroke volume [10,11]. The increase in heart rate associated with heat stress is primarily the result of autonomic innervation, although some studies have indicated that a minor component of this response may be attributable to direct heating of the heart [12]. Furthermore, systolic cardiac function is reportedly influenced by body temperature [13].

In our previous study, using a water-perfused suit, we observed differential cardiac responses to whole-body heat stress between healthy individuals and patients with CSCI with impaired or significantly reduced sympathetic innervation of the heart. We found that the cardiac sympathetic nervous system contributes to systolic cardiac function during hyperthermia [14]. In contrast to the aforementioned whole-body heat stress, head-out hot water immersion (HHWI) is a straightforward method for inducing heat stress and has a long history of use as a hot spring therapy in Japan. Although the impact of HHWI on circulation and cardiac function has been documented in healthy, non-disabled individuals, there is a shortage of research examining the effects of HHWI in individuals with CSCI. In HHWI, both heat and hydrostatic pressure are applied to the body’s surface, resulting in a different type of strain on the heart than that observed during whole-body heat stress using a water-perfused suit. Should the effects and mechanisms of HHWI on the cardiac function of patients with CSCI be elucidated, there is the possibility that this thermal therapy could be applied as a countermeasure for arteriosclerosis and heart disease in patients with CSCI. This study aimed to examine the impact of whole-body heat stress using HHWI on cardiac function in individuals with CSCI and healthy participants and gather fundamental data for thermal therapy using HHWI in individuals with CSCI.

## 2. Materials and Methods

### 2.1. Participants

The study population comprised eight patients with CSCI. Nine healthy subjects of the same sex, age, and height were included as controls. Patients were excluded if they had any comorbid neurological or medical conditions or if they were taking medications that affected cardiac function. Participants provided written informed consent after the purpose and risks of the study were explained in detail. The Ethics Committee of Wakayama Medical University approved the research protocol and consent forms (No. 1279). The study was conducted in accordance with the ethical standards of the Declaration of Helsinki.

The experimental protocol required the participants to abstain from alcohol and caffeine consumption and exercise for 24 h before the experiment. In the laboratory, a probe for deep-body temperature measurement was first inserted into the esophagus from the nasal cavity of each participant at a distance equivalent to one-fourth of the participant’s height. Thereafter, heart rate and blood pressure were checked for stability. After 30 min of rest in a sitting position in a room maintained at 28 °C, the participants were immersed up to the neck in warm water 2 °C above their deep body temperature for 60 min. Thereafter, the participants were placed in a resting position in a room maintained at 28 °C. Subsequently, the participants were positioned in a seated posture and permitted to rest in a room maintained at 28 °C. For patients with CSCI, a lift was employed to facilitate HHWI. The trunk was secured to a chair with a belt to ensure that the participant maintained a sitting position in the water. Furthermore, in order to prevent any effects on cardiovascular responses other than heat stress, the subjects were not provided with any water during HHWI.

### 2.2. Measurements

The esophageal temperature and heart rate were recorded at 50 Hz using an electro-cardiograph and a data acquisition system (Biopac System, Santa Barbara, CA, USA). Furthermore, an automatic arterial manometer was attached to the right middle finger for intermittent measurement of arterial pressure. The data were averaged over the final 60 s of the resting period and the HHWI. Any possible adverse events were investigated.

### 2.3. Echocardiography

Echocardiographic measurements were performed using a commercially available ultrasound system (LOGIQ7, GE Healthcare, Tokyo, Japan). Measurements were taken during expiration in a seated position at the conclusion of the resting period and at the conclusion of the HHWI. To ensure the integrity of the underwater measurements, a combination of vinyl, waterproof film, and cloth tape was employed to maintain the dryness of the probe, except for the tip. The images were captured by two experienced physicians, stored on a hard disk, exported, and analyzed offline by an experienced ultrasound technician using commercially available software (LOGIQ7, GE Healthcare, Tokyo, Japan). The means of the measurements obtained from four consecutive cardiac cycles at the end of rest and HHWI were calculated.

### 2.4. Mitral Inflow Velocities

For echocardiographic measurements, a four-lumen image was obtained, and the left ventricular inflow waveform was recorded using pulse Doppler with a sample volume of 2.0 mm placed over the mitral valve leaflet. The peak inflow velocity was calculated as the early diastolic left ventricular inflow velocity (E) and the late diastolic left ventricular in-flow velocity (A), where E is an index of left ventricular diastolic function and A represents the left atrial contractility.

### 2.5. Tissue Doppler Imaging

Mitral annular velocity, which is less sensitive to left ventricular volume, was measured. A four-lumen image was obtained, and the waveform of the mitral annulus tissue was recorded by tissue Doppler imaging with a 4.0 mm sample volume placed at the junction of the septal and left ventricular walls of the mitral annulus and at the junction of the lateral free wall and the left ventricular wall. The early diastolic mitral annular velocity (E′), late diastolic mitral annular velocity (A′), systolic mitral annular velocity (S′), and systolic mitral annular acceleration (IVA) were calculated [15].

The IVA was calculated from the slope of the presystolic velocity curves at the septal and free walls, lateral to the mitral annular region. The aforementioned indices were calculated for the septal and lateral free walls of the mitral annulus, and mean values were determined. E′ and IVA are indices of left ventricular diastolic function, A′ of left atrial contractility, and S′ and IVA are indices of left ventricular contractility.

### 2.6. Data Analysis

A post hoc power analysis was conducted, which determined that our findings were powered to detect a difference in core temperature for control individuals (power = 100%; alpha = 0.05). A two-sample *t*-test was employed to test differences in age, height, weight, and BMI between the CSCI and control participants groups at baseline, as well as in body temperature, pulse, and blood pressure between the end of the rest period and at the end of HHWI. The Shapiro–Wilk test was used to ascertain the distribution normality of cardiac function variables; for variables that exhibited a normal distribution, a *t*-test was employed. For variables that did not display a normal distribution, a non-parametric test (Wilcoxon test) with paired observations before and after was utilized in each group. All values were expressed as mean ± SD, and *p*-values < 0.05 were considered to indicate a statistically significant difference.

## 3. Results

### 3.1. Participants

This study included nine healthy control participants and eight patients with CSCI. There was no significant difference in age or height between the two groups; however, the weight of the control participants was significantly greater (Table 1 and Table 2). 

Patients with CSCI had injuries to the cervical spinal cord at the C5 level in two cases, the C6 level in four cases, and the C7 level in two cases. All patients with CSCI had complete injuries (A on the American Spinal Injury Association Impairment Scale). The mean time since injury was 9.7 years, and no complications required treatment. Before the heat stress intervention, no significant differences were observed in thermoregulatory and hemodynamic variables between the two groups. Specifically, there were no significant differences in deep body temperature or heart rate. The mean blood pressure was significantly lower in the CSCI group prior to the onset of heat stress. No participants exhibited signs of dehydration.

### 3.2. Cardiac Function Indices

No significant differences were observed in E, E′, A, A′, S′, and IVA between the two groups prior to HHWI.

### 3.3. Thermoregulatory and Hemodynamic Responses

Thermoregulatory and hemodynamic parameters exhibited notable differences between the two groups. Deep body temperature significantly increased from 37.3 ± 0.4 °C to 39.4 ± 0.4 °C in healthy participants and from 37.3 ± 0.5 °C to 39.8 ± 0.7 °C in the CSCI group (Table 2) from before to after HHWI. Despite the significant difference in BMI between the two groups, no difference was evident in the extent of body temperature increase. The heart rate of the control group significantly increased from 75.6 ± 9.2 to 112.9 ± 11.8 bpm, while the CSCI group demonstrated a non-significant increase from 76.4 ± 13.7 to 89.5 ± 11.0 bpm (Table 3). The change in heart rate exhibited by the control group was significantly more pronounced. The mean blood pressure of the control group increased from 93.1 ± 16.4 to 85.7 ± 5.9 mmHg, whereas that of the CSCI group demonstrated a similar rise from 71.6 ± 15.7 to 77.5 ± 9.2 mmHg, indicating no differences in the change in blood pressure between groups (Table 3). The mean blood pressure observed before the administration of heat stress was significantly lower in the CSCI group. No adverse events were reported.

Cardiac function indices significantly increased in the control group, with E (67.0 ± 22.6 to 89.1 ± 13.6 cm/s) and E′ (9.5 ± 3.8 to 15.1 ± 4.1 cm/s) values showing significant increases after HHWI. No significant changes were observed in the CSCI group (Table 4). No notable discrepancies were identified between the two groups. A (50.0 ± 15.2 to 75.8 ± 18.2 cm/s) and A′ (8.1 ± 1.6 to 14.8 ± 5.9 cm/s) showed significant increases after HHWI in the control group. In the group with CSCI, A′ (9 ± 2.4 to 10.8 ± 2.3 cm/s) remained unchanged, whereas A (49.5 ± 9.9 to 56.9 ± 10.9 cm/s) exhibited a significant increase (Table 4). Significant intergroup differences were observed following HHWI. The data indicate a statistically significant increase in S′ (8.7 ± 1.4 to 15.1 ± 4.5 cm/s) and IVA (104.2 ± 14.7 to 151.1 ± 20.6 cm/s^2^) in the control group with the application of heat stress. Only the IVA (94.4 ± 27.2 to 134.7 ± 27.7 cm/s^2^) exhibited a statistically significant increase in individuals with CSCI (Table 4). No significant differences were observed between groups.

## 4. Discussion

The most significant finding of this study was that 60 min of whole-body heat stress with HHWI maintained blood pressure by increasing the heart rate, left atrial contractility, left ventricular diastolic capacity, and left ventricular contractility in healthy participants. In contrast, despite the increased heart rate, left ventricular diastolic function and blood pressure did not change in patients with CSCI. It is plausible that the sympathetic nervous system disorder observed in CSCI patients may have contributed to the left ventricular diastolic dysfunction.

The present study demonstrated that immersing the entire body in warm water (2 °C higher than esophageal temperature) for a period of 60 min resulted in a notable elevation in esophageal temperature in both the control and SCSI groups. This method was deemed to be efficacious as a whole-body warming therapy. Moreover, despite the weight and BMI discrepancy between the two groups, the magnitude of the change in temperature was similar between groups, indicating that body size does not influence the warming effect of this thermal therapy. One of the effects of heat stress on the cardiovascular system is a reduction in venous return to the heart owing to peripheral vasodilation during whole-body heat stress. This has been observed in healthy participants in multiple studies [8,10,16,17]. The contractile function of the left atrium and ventricle increases to maintain or increase the cardiac output and blood pressure owing to decreased venous return [10]. Moreover, HHWI is less likely to induce a reduction in venous return due to hydrostatic pressure. This study demonstrated that whole-body heat stress using HHWI resulted in increased left atrial and ventricular systolic function and left ventricular diastolic function, along with an increase in heart rate, while blood pressure remained unaltered in healthy participants. In contrast, patients with CSCI exhibited an elevated heart rate; however, the enhancements in left atrial function and left ventricular diastolic function observed in healthy participants were not evident in patients with CSCI. However, it is noteworthy that IVA, a measure of ventricular function, significantly increased both in patients with CSCI and in healthy participants. It was postulated that an increase in central venous volume resulting from HHWI might conversely lead to an increase in blood pressure, given that stroke volume did not decline during heat stress in the aforementioned study despite the absence of an increase in cardiac function. Posture may have been a contributing factor, as a blood shift is commonly observed in the sitting position.

The mechanism of whole-body heat stress in the cardiovascular system involves the sympathetic nervous system, vascular function, and neuroendocrine factors, as exemplified by the renin–angiotensin–aldosterone and diuretic peptide systems. In a previous study, the authors focused on the sympathetic nervous system and assessed the impact of sympathetic innervation of the heart on cardiac function during hyperthermia by comparing whole-body heat stress using a circulatory suit in patients with CSCI and thoracolumbar SCI who lacked sympathetic nervous system function and in normal individuals [14]. They found that left atrial and ventricular contractility remained unaltered in patients with CSCI, whereas a notable increase was observed in healthy participants and in those with thoracolumbar SCI. Additionally, left ventricular diastolic capacity was enhanced in all groups. These findings indicate that the sympathetic nervous system plays an important role in the increase in cardiac left atrial and ventricular systolic functions caused by whole-body heat stress using a circulatory suit. Additionally, they suggested that another factor influences left ventricular diastolic performance. This study aimed to evaluate whole-body heat stress in patients with CSCI using HHWI with the aim of developing a more convenient thermal therapy. The results demonstrated that heart rate increased in a manner comparable to that observed in the control group. However, no significant change was noted in left ventricular diastolic function in patients with CSCI, whose sympathetic nervous system was not functioning. However, A, an indicator of left atrial contractility, and IVA, an indicator of ventricular function, exhibited a notable increase in the CSCI group, aligning with the observations made in the control group. Additionally, blood pressure remained stable throughout the experiment. The results demonstrated that the application of a circulatory suit and HHWI in conjunction with a whole-body heat load had disparate effects on left atrial contractility, left ventricular contractility, and left ventricular diastolic function in patients with CSCI. It was hypothesized that the reason for this discrepancy was that, in addition to heating, HHWI also involves water pressure on the body surface. This may have resulted in a different cardiac load compared to that of the whole-body heat load using a circulatory suit. Additionally, the difference in venous return to the heart due to the difference in posture between lying down and sitting may have contributed to this phenomenon. Furthermore, it was hypothesized that the sympathetic nervous system played a pivotal role in enhancing left ventricular diastolic function after HHWI. Additionally, there is a possibility that other factors, such as the direct thermal effect on the heart, vascular function, the renin–angiotensin–aldosterone system, and the diuretic peptide system, may also have influenced left atrial and left ventricular contractility. Individuals with SCSI exhibit a diminished activity level and basal metabolism due to paralysis. Consequently, they are at an elevated risk of developing lifestyle-related diseases, including arteriosclerosis, hyperlipidemia, hypertension, elevated blood sugar, insulin resistance, and cardiovascular disease in comparison to those who are healthy. It has been demonstrated that exercise therapy is an effective method for the prevention of disease [18]. The thermal therapy utilized in this study can be performed in the domestic setting, specifically in a bath, and has the potential to be an efficacious thermal therapy not only in a hospital rehabilitation context but also in everyday life. This study should be regarded as a foundation for further research in this field. Furthermore, it has been demonstrated that cardiac rehabilitation enhances ventricular contractility and dilatation in patients with heart disease [19,20]. Given that HHWI augmented the ventricular contractility of patients with CSCI and healthy individuals in this investigation, HHWI may serve as a potential cardiac rehabilitation strategy for these patients.

The present study is limited by its small sample size and the fact that all subjects were male. Additionally, the analysis is limited to a single heat stress session, with fixed temperature and time. Consequently, the changes that occurred during the load were not examined. Moreover, the findings of this study are potentially influenced by confounding factors beyond the heat stress itself.

## 5. Conclusions

In this study, we subjected healthy individuals and patients with CSCI to whole-body heat stress using HHWI and observed that the effect of the increase in deep body temperature was equivalent in both groups. Furthermore, the procedure was safe and did not result in any changes in blood pressure. Additionally, left atrial contractility, left ventricular dilatation capacity, and left ventricular contractility improved in healthy participants, while in patients with CSCI, left atrial contractility and left ventricular contractility improved, but there was no improvement in left ventricular diastolic function. The findings of this study suggest that the sympathetic nervous system plays a primary role in left ventricular diastolic function. Additionally, other factors may potentially contribute to left atrial contractility and left ventricular contractile function. It is our contention that this research will provide crucial fundamental data for the implementation of whole-body hyperthermia using neck immersion as a prophylactic measure against complications such as lifestyle-related diseases in patients with CSCIs.

## Figures and Tables

**Table 1:** Physical characteristics of groups.

Group	Age (Year)	Height (cm)	Weight (kg)	Level of Spinal Lesion
AB-1	31	179.2	73.7	
AB-2	33	165.9	65.2	
AB-3	49	169.6	78.1	
AB-4	52	166	70.3	
AB-5	42	179.8	69.8	
AB-6	49	167	86.2	
AB-7	34	176	67.9	
AB-8	38	168	58.8	
AB-9	29	182	68.6	
CSCI-1	49	157.3	57.9	C7
CSCI-2	39	168.4	60	C7
CSCI-3	58	159	42.3	C5
CSCI-4	31	178	59.9	C5
CSCI-5	29	168	56.2	C6
CSCI-6	23	180	60.75	C6
CSCI-7	41	163	38.75	C6
CSCI-8	35	181	69.2	C6

Abbreviations: AB, abled body; CSCI, cervical spinal cord injury.

**Table 2:** Comparison of physical characteristics between AB and CSCI groups.

	AB (n = 9)	CSCI (n = 8)	*p* Values
Age (year)	39.6 ± 8.5	38.0 ± 11.3	0.755
Heigh (cm)	172.6 ± 6.6	169.2 ± 9.6	0.414
Weight (cm)	71.0 ± 7.8	55.6 ± 10.1	0.0031 *
Body mass index (kg/m^2^)	23.9 ± 3.4	19.3 ± 2.8	0.0083 *
Level of spinal lesion			
C5		2	
C6		4	
C7		2	
Classification ASIA		A	
Time since injury(year)		9.7 ± 7.2	

Abbreviations: AB, abled body; CSCI, cervical spinal cord injury; ASIA, American Spinal Injury Association. Significance * *p* <0.05.

**Table 3:** Results of core temperature, heart rate and mean blood pressure.

	Rest	Post Immersion	*p* Values
			(Rest vs. Post)
Core temperature (°C)			
AB	37.3 ± 0.4	39.4 ± 0.4	<0.0001 *
CSCI	37.3 ± 0.5	39.8 ± 0.7	<0.0001 *
*p* values (AB vs. CSCI)	0.83228	0.2105	
Heart rate (bpm)			
AB	75.6 ± 9.2	112.9 ± 11.8	<0.0001 *
CSCI	76.4 ± 13.7	89.5 ± 11.0	0.0023 *
*p* values (AB vs. CSCI)	0.8857	0.0008 *	
Mean blood pressure (mmHg)			
AB	93.2 ± 16.4	85.7 ± 5.9	0.2177
CSCI	71.6 ± 15.7	77.5 ± 9.2	0.3765
*p* values (AB vs. CSCI)	0.0146 *	0.0413 *	

Abbreviations: AB, abled body; CSCI, cervical spinal cord injury. Significance * *p* < 0.05.

**Table 4:** Results of cardiac function.

	Rest	Post Immersion	*p* Values
			(Rest vs. Post)
E			
AB	67.0 ± 22.6	89.1 ± 13.6	0.0078 *
CSCI	73.2 ± 25.2	81.5 ± 22.2	0.25
*p* values (AB vs. CSCI)	0.5986	0.3989	
E′			
AB	9.5 ± 3.8	15.1 ± 4.1	0.0117 *
CSCI	11.4 ± 4.0	13.2 ± 5.3	0.641
*p* values (AB vs. CSCI)	0.3118	0.4306	
S′			
AB	8.7 ± 1.4	15.1 ± 4.5	0.0039 *
CSCI	10.8 ± 3.3	12.0 ± 4.0	0.547
*p* values (AB vs. CSCI)	0.105	0.1593	
IVA			
AB	104.2 ± 14.7	151.1 ± 20.6	0.0039 *
CSCI	94.4 ± 27.2	134.7 ± 27.7	0.0078 *
*p* values (AB vs. CSCI)	0.3636	0.1813	
A			
AB	50.0 ± 15.2	75.8 ± 18.2	0.0039 *
CSCI	49.5 ± 9.9	56.9 ± 10.9	0.0039 *
*p* values (AB vs. CSCI)	0.9423	0.0217 *	
A′			
AB	8.1 ± 1.6	14.8 ± 5.9	0.0039 *
CSCI	9.0 ± 2.4	10.8 ± 2.3	0.25
*p* values (AB vs. CSCI)	0.3822	0.089	

AB, abled body; CSCI, cervical spinal cord injury; E, mitral inflow velocity during the early phase of left ventricular relaxation; E′, early diastolic mitral annular velocity; S′, mitral annular systolic velocity; IVA, isovolumic acceleration; A, mitral inflow velocity during left atrial contraction; A′, late diastolic mitral annular velocity. Significance * *p* < 0.05.

## Data Availability

There are no data available to describe.
